# The Intestine as a Lifespan- and Proteostasis-Promoting Signaling Tissue

**DOI:** 10.3389/fragi.2022.897741

**Published:** 2022-06-02

**Authors:** Francesca Hodge, Viktoria Bajuszova, Patricija van Oosten-Hawle

**Affiliations:** Astbury Centre for Structural Molecular Biology, Faculty of Biological Sciences, School of Molecular and Cellular Biology, University of Leeds, Leeds, United Kingdom

**Keywords:** intercellular signaling, intestine, cell-nonautonomous, proteostasis, *C. elegans*, stress, organismal aging, neurons

## Abstract

In multicellular organisms such as *Caenorhabditis elegans*, cellular stress stimuli and responses are communicated between tissues to promote organismal health- and lifespan. The nervous system is the predominant regulator of cell nonautonomous proteostasis that orchestrates systemic stress responses to integrate both internal and external stimuli. This review highlights the role of the intestine in mediating cell nonautonomous stress responses and explores recent findings that suggest a central role for the intestine to regulate organismal proteostasis. As a tissue that receives and further transduces signals from the nervous system in response to dietary restriction, heat- and oxidative stress, and hypoxia, we explore evidence suggesting the intestine is a key regulatory organ itself. From the perspective of naturally occurring stressors such as dietary restriction and pathogen infection we highlight how the intestine can function as a key regulator of organismal proteostasis by integrating insulin/IGF-like signaling, miRNA-, neuropeptide- and metabolic signaling to alter distal tissue functions in promoting survival, health- and lifespan.

## Introduction

Efficient protein folding is essential for the vitality of all cells within an organism throughout the different challenges that occur in a lifetime. Maintaining a functional proteome is therefore crucial for cellular health in all living organisms. This is achieved by the cellular proteostasis network, and predominantly enacted by molecular chaperones as well as degradation machineries such as the proteasome and autophagy [reviewed in Hipp et al. (2019)]. In order to combat protein misfolding and environmental fluctuations, multicellular organisms require a coordinated response mounted between tissues. Recognition of this led to a shift in focus towards investigations into the cell nonautonomous regulation of proteostasis and how it impacts both health- and lifespan in normal and pathological aging [reviewed in O’Brien and van Oosten-Hawle (2016); reviewed in Sala et al. (2017); reviewed in Hipp et al. (2019); reviewed in Morimoto (2020)]. Recent evidence suggests that cell nonautonomous regulation of proteostasis is a highly complex process with different outcomes based on the tissue experiencing the stress (Taylor and Dillin, 2013; van Oosten-Hawle et al., 2013; Shao et al., 2016).

A crucial component to regulate organismal proteostasis is the cell nonautonomous control of stress responses such as the unfolded protein response of the ER (UPR^ER^) (Lee, 2021), the unfolded protein response of the mitochondria (UPR^MIT^) [reviewed in Jovaisaite et al. (2014)], the heat shock response (HSR) (Prahlad et al., 2008) and transcellular chaperone signaling (TCS) (van Oosten-Hawle et al., 2013). Throughout the aging process the competency of stress responses are known to decline in *C. elegans* [Ben-Zvi et al. (2009); reviewed in; Labbadia and Morimoto (2014); Kim et al. (2018); reviewed in; Muñoz-Carvajal and Sanhueza (2020)] which often correlates with the onset of age-related diseases such as Alzheimer’s (AD) [reviewed in Campanella et al. (2018)] and Huntington’s disease (HD), as shown in *C. elegans* models (Labbadia et al., 2011) and mouse models (Wang et al., 2008), as well as in the human pathology; for example, HSF1 is degraded in brain samples of HD patients (Gomez-Pastor et al., 2017; Koyuncu et al., 2018). Enhancing and “hijacking” cellular stress responses in animals modeling these disease states can be beneficial in delaying or even preventing age-associated pathologies [reviewed in Ben-Zvi et al. (2009); Vidal et al. (2012); Bakula and Scheibye-Knudsen (2020)] which is highlighted and described in detail elsewhere in this special issue.

Many of the cell nonautonomous stress responses are controlled by the nervous system, in both *C. elegans* as well as vertebrate models (Bishop and Guarente, 2007; Durieux et al., 2011; Taylor and Dillin, 2013; Williams et al., 2014; Shao et al., 2016), opening new questions on the exact neural circuits orchestrating organismal proteostasis. Beyond the nervous system the intestine has been shown to not only integrate stress signals received from the neurons but is yet another organ central for the regulation of proteostasis. This review will explore the role of the intestine as a proteostasis-regulating tissue and the consequences for organismal health- and lifespan with an emphasis on findings from the model organism *C. elegans*.

## The Integration of Nervous System Signals to the Intestine to Promote Organismal Proteostasis and Lifespan

**FIGURE 1:**
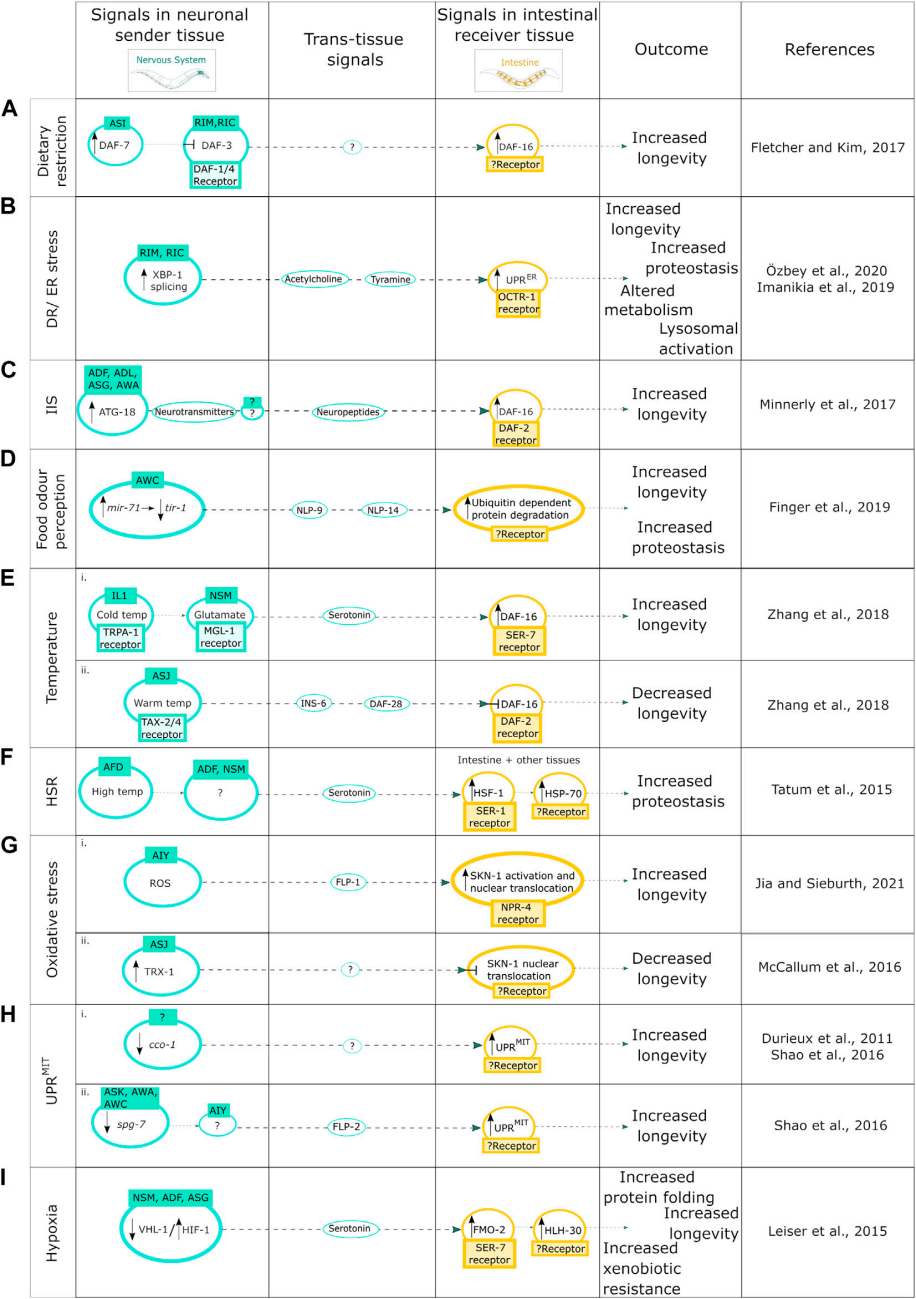
Neuron-to-intestine communication in organismal proteostasis and aging. **(A)** Mechanism of DR induced neuronal cell nonautonomous signaling upon expression of DAF-7. **(B)** DR/ER stress-induced neuronal cell nonautonomous signaling upon activation of the IRE-1-XBP-1 branch of the UPR^ER^. **(C)** Neuronal cell nonautonomous signaling upon increase of the autophagy protein ATG-18; **(D)** upon food odor perception through the miRNA pathway; **(E)** in response to (i) cold temperature and (ii) high temperature; **(F)** in response to the HSR; **(G)** in response to oxidative stress; induced by (i) ROS, (ii) TRX-1 activation; **(H)** upon induction of neuronal UPR^MIT^ by the KD of the ETC component (i) *cco-1* and (ii) *spg-7*; and **(I)** in response to hypoxia.

Cell nonautonomous neuroendocrine signaling pathways, such as those which initiate trans-tissue communication from the olfactory neurons to the intestine, are important for *C. elegans* health and lifespan and have been shown to regulate proteotoxic stress and quality control [reviewed in Miller et al. (2020)]. For example, chemosensory neurons send neuroendocrine signals in response to food cues, such as low food quantity, also known as dietary restriction (DR), to regulate the activity of the FOXO transcription factor DAF-16/FOXO in the intestine, the main component of the insulin-like signaling (IlS) pathway, that regulates longevity (Fletcher and Kim, 2017). DR induces the expression of the neuroendocrine ligand DAF-7 in the ASI chemosensory neurons, which signal to the RIM and RIC interneurons to suppress the co-SMAD DAF-3*.* This enables the induction of the DR response and the activation of DAF-16 in the intestine to promote longevity (Fletcher and Kim, 2017). However, in aging nematodes, DAF-7 expression levels are decreased and thus are no longer inhibiting DAF-3*,* which reduces the capacity for DR-induced lifespan extension in older animals (Fletcher and Kim, 2017) (Figure 1A).

Furthermore, DR, as well as the induction of ER stress *via* neuronal overexpression of *xbp-1s*, can activate the neuronal IRE-1-XBP-1 branch of the UPR^ER^ (Matai et al., 2019; Özbey et al., 2020), a stress-response pathway counteracting unfolded protein stress in the endoplasmic reticulum, that is, involved in lifespan regulation [reviewed in Taylor and Hetz (2020); reviewed in Walter and Ron (2011)]. DR can induce the splicing of XBP-1 into XBP-1s in the RIM and RIC neurons which drives cell nonautonomous UPR^ER^ activation in the intestine *via* acetylcholine and tyramine signaling leading to metabolic and lysosomal changes, and lifespan- and proteostasis-enhancing effects on an organismal level (Imanikia et al., 2019a, 2019b; Özbey et al., 2020) (Figure 1B).

In addition, DR as well as reduced IlS signaling (as modelled *via* the use of *daf-2* mutants) can cause an increase in the autophagy protein ATG–18 activity in chemosensory neurons, and the intestine, resulting in lifespan extension in a cell nonautonomous manner (Minnerly et al., 2017). The exact cell nonautonomous mechanism of the ATG-18 mediated lifespan extension upon DR is not yet fully known, however in loss of function *daf-2* mutants an increased activity of ATG-18 in ADF, ADL, ASG, and AWA neurons sends a signal through neurotransmitters to an unknown neuron which in turn signals to the intestine *via* neuropeptides to activate DAF-16 and thus increase longevity (Minnerly et al., 2017) (Figure 1C).

Interestingly the perception of food odor itself can maintain organismal proteostasis and regulate lifespan through the microRNA *miR-71* mediated inhibition of *tir-1* mRNA stability in olfactory AWC neurons (Finger et al., 2019) (Figure 1D). Importantly, AWC neuron activity has a direct impact on ubiquitin-dependent protein degradation in the intestine, which is regulated *via* secretion of the neuropeptides NLP-9 and NLP-14, leading to increased longevity and proteostasis (Finger et al., 2019) (Figure 1D).

Efficient communication between the nervous system and the intestine is also required for effective survival upon exposure to cold and warm temperatures (Zhang et al., 2018). Under both circumstances, neurons require DAF-16 activity specifically within the intestine to integrate cues from the environment as well as the nervous system to impact longevity under different temperature conditions (Zhang et al., 2018). For example, low temperature sensing IL1 and NSM neurons send signals to the intestine to extend lifespan through glutamate and serotonin neurotransmitters, whereas the warm temperature sensing ASJ neurons send signals to the intestine through insulin-like neuropeptides to shorten lifespan (Zhang et al., 2018) (Figure 1E). These opposing effects on longevity are *via* differential regulation of the transcription factor DAF-16 where IL1 and NSM neurons send signals to activate DAF-16 expression (Figure 1Ei) whereas ASJ neurons send signals to inhibit DAF-16 expression in the intestine (Zhang et al., 2018) (Figure 1Eii). In addition, exposure to high temperatures activates the HSR, which has proteostasis and longevity promoting effects (Morley and Morimoto, 2004). In *C. elegans*, thermo-sensory AFD neurons respond to an increase in temperature by regulating the cell nonautonomous HSR (Prahlad et al., 2008) by activating the heat shock transcription factor heat shock factor-1 (HSF-1), in distal tissues including the intestine (Tatum et al., 2015). This cell nonautonomous HSF-1 activation is mediated *via* the release of serotonin from ADF and NSM neurons and activation of the SER-1 receptor and results in induction of heat shock proteins such as HSP-70 that facilitates the reduction of protein misfolding (Tatum et al., 2015) (Figure 1F).

**FIGURE 2:**
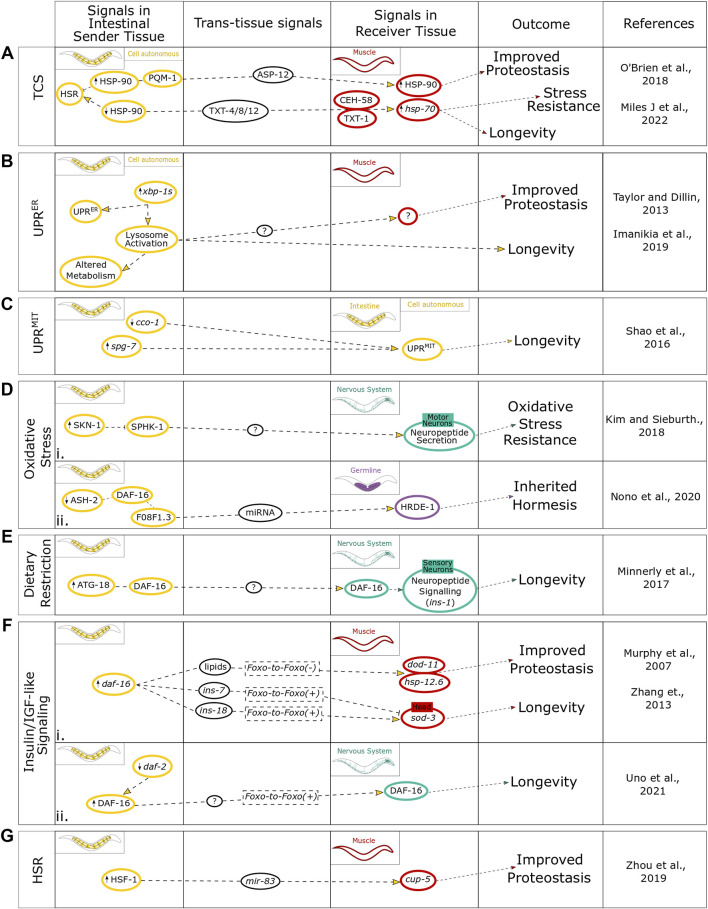
Summary of intestinal regulation of cell nonautonomous stress signals in mediating organism physiology. The findings for intestine-induced stresses within the context of **(A)** TCS cell autonomously and communicated to the muscle, **(B)** the UPR^ER^ cell autonomously and communicated to the muscle, **(C)** the UPR^MIT^ contained to a cell autonomous response, **(D)** oxidative stress communicated to both (i) the nervous system and (ii) the germline, **(E)** dietary restriction communicated to the nervous system, **(F)** IlS communicated to both (i) the muscle and (ii) the nervous system, and **(G)** the age-associated changes in the competency of the HSR. This figure highlights the signals within the intestinal sender tissue, signals involved in the trans-tissue communication, and signals within the different receiver tissues and how they link to different physiological outcomes.

Integration of neuronal signals by the intestine also plays an important role in the response to oxidative stress (Kim and Sieburth, 2018). Reactive oxygen species (ROS), which are the main cause of oxidative stress, can promote premature and pathological aging, however, they can also increase organismal stress resistance and longevity, thus having a dual effect [reviewed in Miranda-Vizuete and Veal (2017)]. The ROS-induced oxidative stress response can be activated by the neuroendocrine stress signal, FLP-1, which upon secretion from the AIY neurons activates the antioxidant response in the intestine, and positively regulates the oxidative stress response transcription factor SKN-1 to modulate lifespan (Jia and Sieburth, 2021) (Figure 1Gi). SKN-1, however, also has a redox-independent role through cell nonautonomous regulation *via* the thioredoxin, TRX-1, that is, expressed in ASJ neurons and suppresses the cell nonautonomous nuclear localization of SKN-1 in the intestine through the p38 MAPK pathway (McCallum et al., 2016) (Figure 1Gii). Neuronal ROS can also induce cell nonautonomous activation of intestinal UPR^MIT^, however this cell nonautonomous activation can also be induced *via* the knockdown of mitochondrial components (Durieux et al., 2011; Shao et al., 2016). Both the reduction in activity of the complex IV subunit *cco-1* and the knockdown of the mt-AAA protease *spg-7* in the intestine induce cell nonautonomous activation of the UPR^MIT^ leading to an increased lifespan (Figure 2C) (Durieux et al., 2011; Shao et al., 2016). The signal/mediator of cell nonautonomous UPR^MIT^ induction *via cco-1* knockdown in either neurons or intestine is not yet known (Durieux et al., 2011; Shao et al., 2016) (Figure 1Hi). The cell nonautonomous induction of UPR^MIT^, when induced by neuronal *spg-7* knockdown, is mediated *via* the secretion of the neuropeptide FLP-2 from AIA interneurons (Shao et al., 2016) (Figure 1Hii). It will be interesting to investigate whether similar or the same mediating components regulate the cell nonautonomous UPR^MIT^ upon *cco-1* knockdown.

Another cellular stress response pathway that requires cell nonautonomous neuron-to-intestine signaling is the hypoxic stress response. Chronic low levels of oxygen induce the hypoxic stress response, regulated *via* the hypoxia-inducible factor (HIF-1), which drives cytoprotective mechanisms in response to this stress and modulates neural circuit function and activity (Pender and Horvitz, 2018). Activation of HIF-1 has been shown to increase the expression of serotonin in NSM, ADF, and ASG sensory neurons (Pocock and Hobert, 2010). Increased pan-neuronal expression of HIF-1 in neurons activates increased production of the longevity gene FMO-2 in the intestine through serotonergic signaling and the SER-7 receptor, which in turn activates the induction of the transcription factor HLH-30 to increase longevity (Leiser et al., 2015) (Figure 1I). Interestingly, intestinal FMO-2 induction can also be activated *via* DR, however, this is potentially regulated through a different signaling pathway (Leiser et al., 2015).

Overall, neuron-to-intestine cell nonautonomous signaling has a crucial role in the pro-longevity actions through dietary restriction, ER stress, IlS, food odor perception and *via* the temperature-, heat shock-, oxidative-, and hypoxic stress responses.

## The Intestine as a Regulating and Integrating Organ for Organismal Proteostasis

While the intestine is an important organ for the integration of neuronal signals to benefit organismal proteostasis, it can potentially act independent of neuronal input. An example of this is that HSP-90 overexpression solely in the intestine is able to communicate *via* the transcription factor PQM-1 and the extracellular immune-peptide ASP-12 to upregulate HSP-90 in the neighboring muscle cells (O’Brien et al., 2018). The subsequent muscular HSP-90 upregulation is able to suppress the age-dependent aggregation of amyloid beta (Aβ) in the muscle (O’Brien et al., 2018). Conversely, intestinal *hsp-90* knockdown has been found to signal *via* secreted peptides TXT-4, TXT-8, and TXT-12 to induce the guanylate cyclase TXT-1 and the transcription factor CEH-58 in muscle cells leading to upregulation of *hsp-70* which results in an improved survival when exposed to elevated temperatures (Miles et al., 2022). These findings provide evidence that the intestine can be a major regulator of proteostasis and stress resistance through cell nonautonomous regulation of molecular chaperones *via* transcellular chaperone signaling (TCS) (Figure 2A) (O’Brien et al., 2018). Intestinal induction of the UPR^ER^ through expression of *xbp-1s* led to a modest increase in lifespan and reduced Aβ aggregation in muscle cells. However, the intestine by itself appears to be unable to induce an UPR^ER^ stress response in distal tissues (Figure 2B) (Taylor and Dillin, 2013). Intercellular activation of the UPR^ER^ from one organ to another specifically requires neuronal expression of *xbp-1s,* with just two interneurons triggering the activation of the UPR^ER^ to distal tissues by tyramine (Özbey et al., 2020). While intestinally induced UPR^ER^ remains cell autonomous, the nervous system requires transcellular activation of the UPR^ER^ in the intestine as an intermediate signaling tissue to produce the organismal benefits on longevity and proteotoxicity, potentially due to intestinal activation of lysosomal factors including *rde-1*, *lmp-1*, *vha-18*, and *asp-3* that exert these beneficial effects in distal tissues (Figure 2B) (Imanikia et al., 2019a). A comparable finding was put forward for intestinal induction of the mitochondrial UPR, with pan-neuronal induction leading to activation of the UPR^MIT^ in distal tissues, whereas intestinal induction of UPR^MIT^ remained cell autonomous but was still able to enhance longevity (Figure 2C) (Shao et al., 2016). Thus, even though the intestine is unable to transduce the activation of a stress response to distal tissues, as shown by the examples of the UPR^ER^ and UPR^MIT^, it is instrumental to mediate the consequences on proteostasis at an organismal level.

When observing naturally occurring stresses that impact the intestine, such as DR or pathogen infection, the intestine becomes an undeniably major tissue required for the tissue-specific as well as “transcellular” regulation of stress stimuli impacting *C. elegans* health- and lifespan. The importance of the intestine is highlighted through its role in the gut-brain axis in humans where the intestine plays a key role in the regulation of brain health that influences the development of neurodegenerative diseases [reviewed in Houser and Tansey (2017); reviewed in Peterson (2020)].

## Oxidative Stress Responses Regulated by the Intestine

In response to oxidative stress, the intestine has been found to integrate neuronal and environmental cues *via* intestinal expression of the conserved transcription factor SKN-1 (Nrf2 in humans) [reviewed in Blackwell et al. (2015)]. The loss of function of this transcription factor significantly reduces oxidative stress tolerance leading to reduced survival (Bishop and Guarente, 2007). *C. elegans skn-1* null mutants that express wild type SKN-1 solely in the intestine are able to survive oxidative stress, whereas animals expressing SKN-1 solely in neurons remained unable to tolerate oxidative stress with extremely low survival rates (Bishop and Guarente, 2007). Interestingly, the intestine mediates the response to oxidative stress by utilizing the neuropeptide network *via* motor neurons (Figure 2Di) (Kim and Sieburth, 2018). Similarly, intestinal IlS regulates resistance to oxidative stress, with knockdown of *daf-2* in the intestine increasing oxidative stress resistance in a *daf-16* dependent manner (Uno et al., 2021). This demonstrates the importance of the intestine as a central orchestrating organ, with the capacity to control proteostasis, perhaps *via* feedback signaling to neural circuits. Oxidative stress is also an important factor in the immune response upon pathogen infection affecting the intestine. For example, *C. elegans* can produce ROS within the intestine upon infection with pathogenic bacteria, such as *Enterococcus faecalis*, as a means of immune defense (Chávez et al., 2007). While ROS can increase *C. elegans* survival rate infected by pathogenic bacteria, high levels of ROS production is cytotoxic and has the potential to also damage host tissues and organs. This can be counteracted by the upregulation of oxidative stress enzymes including *sod-3* and *clt-2* (Chávez et al., 2007). Beyond their role in pathogen defense, antioxidants also have effects on lifespan, behavior and proteostasis (Brown et al., 2006). For example, *C. elegans* exposed to the antioxidant epigallocatechin gallate (EGCG) *via* feeding reduces the age-associated decline of pharyngeal pumping but did not lead to a lifespan extension (Brown et al., 2006); whereas the antioxidant α-lipoic acid has the opposite effect by increasing lifespan, potentially through differences in specific gene expression with different molecular targets. Both antioxidants are able to improve age-associated decline in chemotaxis ability, indicating roles in influencing neuronal signaling and behavioral consequences (Brown et al., 2006).

In humans, oxidative stress has been linked with the progression of AD, as the gut-brain axis is crucial for brain health [reviewed in Dumitrescu et al. (2018); reviewed in Luca et al. (2019)]. For example, upon a 12-week oral probiotic supplementation program the oxidative stress biomarker ‘malondialdehyde’ was reduced which correlated with an increase in cognition in AD patients (Akbari et al., 2016). Therefore, further characterization of this link between the intestine and nervous system in the context of oxidative stress may provide potential targets for therapeutics in AD.

## Intestinal Regulation of Dietary Restriction

DR is a common stress that *C. elegans* will encounter naturally when food abundance is low. The molecular process by which DR is sensed, interpreted and resolved is considered a complex and incompletely understood process. There is, however, a common consensus that the major tissues perceiving dietary restriction are the neurons and the intestine with information on environmental cues of food abundance interpreted by the nervous system and actual nutritional uptake by the intestine (Walker et al., 2005). A recent study investigated the role of autophagy in the regulation of lifespan through DR as well as the IlS pathway in *C. elegans* (Minnerly et al., 2017)*.* Intestinal expression of autophagy factor ATG-18 is required to respond to DR, enabling DR-mediated longevity by targeting neuropeptide communication in the nervous system (Figure 2E). In parallel, and independent of DR, intestinal expression of ATG-18 also influences the expression of insulin like-peptide *ins-1* in neurons and requires neurotransmitter release to promote longevity *via* the IlS pathway (Minnerly et al., 2017). Importantly, the intestine plays a crucial role for DR-induced life- and health-span extension through increased autophagic flux by alleviating the age-related decline in motility and improving the intestinal barrier function (Gelino et al., 2016). This contribution of autophagy to healthspan extension is largely observed in the genetically dietary restricted *eat-2* mutant, where autophagic flux is increased, resulting in a reduced age-dependent loss of gut integrity (Gelino et al., 2016). Interestingly, in humans, gut dysbiosis also reduces intestinal integrity and can be a source of oxidative stress that contributes to the initiation of neurodegenerative diseases [reviewed in Dumitrescu et al. (2018)].

## Intestinal Regulation of the Insulin/Insulin-Like Growth Factor-Like Signaling Pathway and FOXO-to-FOXO Signaling

The IlS pathway is the predominant pathway integrating different nutritional cues resulting in system-wide impacts through several aspects of healthspan including fertility (Klass, 1977; Tissenbaum and Ruvkun, 1998); reviewed in Neirijnck et al. (2019), stress resistance [reviewed in Yu and Chung (2001)], immunity (Singh and Aballay, 2006) and metabolism [reviewed in Anderson and Weindruch (2007)], as well as having direct impacts on lifespan (Kenyon et al., 1993). The IlS pathway is inherently a cell nonautonomous pathway with activation signals in the form of extracellular insulin-like peptides binding the cell-surface receptor DAF-2/IGFR. This in turn triggers a kinase cascade culminating in the phosphorylation of the main FOXO transcription factor DAF-16 (Ogg et al., 1997) which subsequently translocates to the nucleus leading to a specific transcriptional program promoting longevity and stress resistance (Henderson and Johnson, 2001). Intestinal DAF-16 has been found to control proteostasis and longevity through cell nonautonomous mechanisms which can be either dependent on DAF-16 in receiver tissues [*FOXO-to-FOXO* (*+*) signaling] (Murphy et al., 2007; Uno et al., 2021) or through an alternative mechanism independent of DAF-16 function in distal tissues [*FOXO-to-FOXO* (*-*) signaling] (Murphy et al., 2003; Zhang et al., 2013). In *FOXO-to-FOXO* (+) signaling, intestinal DAF-16 induces an upregulation of the DAF-16-dependent gene *sod-3* in the epidermis and muscle, correlating with an increased lifespan (Figure 2Fi). This intestinal *FOXO-to-FOXO* (*+*) signaling was found to be dependent on the positive and negative regulation of the insulin-like genes *ins-18* and *ins-7*, respectively. In particular, *ins-7* knockdown in the intestine induced *sod-3* expression in head muscles and enhanced lifespan whereas intestinal *ins-18* knockdown prevented *sod-3* expression in head muscles and caused a shortened lifespan creating a positive feedback loop that coordinates system-wide aging (Figure 2Fi) (Murphy et al., 2007). The intestinal-neuronal axis is vital in this cell non-autonomous regulation of lifespan as well as reproductive span, with knockdown of *daf-2* in the gut being sufficient for lifespan extension (Uno et al., 2021). Interestingly, *daf-2* knockdown in the intestine also induced nuclear localization and activation of DAF-16 in the neurons (Figure 2Fii), further suggesting IlS-mediated *FOXO-to-FOXO* (*+*) signaling can be employed from the intestine to the neurons to regulate lifespan.

The intestine has also been found to employ IlS in *FOXO-to-FOXO* (*-*) trans-tissue communication (Figure 2Fi). For example, intestine-specific overexpression of DAF-16 extended the lifespan of *daf-16* and *daf-2* null mutants by up to 70%, suggesting this longevity phenotype is not dependent on DAF-16 in distal tissues (Libina et al., 2003). The intestine was also the only tissue requiring DAF-16 activity to maintain germline-defective induced longevity, suggesting a potential cell nonautonomous connection between the intestine and germline within the context of fitness trade-off. Interestingly, intestinal DAF-16 overexpression was able to upregulate the expression of proteostasis and metabolic genes, such as *hsp-12.6* and *dod-11*, respectively, *via FOXO-to-FOXO* (*-*) signaling (Figure 2Fi), in tissues lacking DAF-16 (Zhang et al., 2013). These *FOXO-to-FOXO* (*-*) signals were found to be dependent on the lipid signal *mdt-15* a subunit of a mediator complex that regulates the expression of genes involved in lipid metabolism, suggesting lipids could be enactors of this pathway. In age-associated protein misfolding disease models, the intestine was able to utilize this *mdt-15*-dependent *FOXO-to-FOXO* (*-*) signaling to improve muscular proteostasis and alleviated the age-dependent paralysis caused by muscular Aβ expression (Zhang et al., 2013). When human insulin is introduced into the *C. elegans* intestine through feeding bacteria supplemented with a buffered suspension of insulin complexed with protamine sulfate, used to treat type II diabetes in humans, this inhibited α-synuclein aggregation in the muscle through antagonizing the DAF-2/IGFR receptor (Haque et al., 2020). This suggests there is some commonality in the functioning of this pathway between humans and *C. elegans* which may be beneficial in neurodegenerative diseases. Low levels of the Insulin-like growth factor 1 (IGF-1) is a risk factor for developing neurodegenerative disease (Westwood et al., 2014) and modulation of IGF-1 by estrogens is thought to cause slower progression of Parkinson’s disease in women compared to men [reviewed in González et al. (2008); reviewed in Labandeira-Garcia et al. (2016); reviewed in Castilla-Cortázar et al. (2020)]. Therefore, deepening our understanding of how to modulate the function of the IlS pathway and what off-target effects this may produce could prove therapeutically beneficial.

## Intestinal Regulation of the MicroRNA Pathway

Another regulatory pathway which is becoming increasingly detailed in its role in trans-tissue communication of stress responses and aging is the microRNA (miRNA) pathway [reviewed in Leung and Sharp (2010); reviewed in Inukai and Slack (2013); reviewed in Son et al. (2019)]. MiRNAs are single-stranded RNAs approximately 22 nucleotides in length which play roles in regulating expression patterns with the ability to either stabilize or destabilize complementary target mRNAs to either increase or decrease translation [reviewed in Fabian et al. (2010)]. The intestine has been implicated in utilizing miRNAs to modulate distal tissue function in promoting health- and lifespan. For example, the age-associated increase of HSF-1 was found to regulate the miRNA *mir-83* specifically within the intestine which impaired intestinal autophagy through targeting a lysosomal calcium channel, *cup-5* (Zhou et al., 2019)*.* This suppression of autophagy by intestine-specific expression of *mir-83* extended to the body wall muscle, by directly impacting the age-associated aggregation of PolyQ (Figure 2G). *mir-83* null mutants showed reduced PolyQ aggregation not only within the intestine but also in the muscle tissue and resulted in an increased lifespan (Zhou et al., 2019). Despite not being directly expressed in muscle, *mir-83* was found to directly regulate *cup-5* transcription in the muscle through interaction with its 3′UTR. Importantly, *mir-83* was detected in both extracellular vesicles and in coelomocytes, suggesting that *mir-83* was transported directly from the intestine to the muscle to enact the cell nonautonomous regulation of autophagy and lifespan.

Aside from autophagy, the intestine has also been found to modulate oxidative stress *via* the miRNA *mir-60* by maintaining cellular homeostasis to promote survival and lifespan (Kato et al., 2016). Intestinal miRNAs are also required for inherited hormesis in response to oxidative stress. When *C. elegans* are exposed to different stresses during developmental stages including osmotic stress, heavy metal stress, and DR this results in increased resistance to oxidative stress which can be passed on to subsequent generations (Kishimoto et al., 2017). Disrupting miRNA transport from the intestine partially suppressed the increased oxidative stress resistance of animals experiencing this as early life stress, but the oxidative stress resistance was completely abolished in their first-generation progeny (Okabe et al., 2021). This suggested that transmission of miRNA from the intestine to the germline may regulate the inheritance of oxidative stress resistance. Further support that the intestine regulates the intergenerational transmission of oxidative stress resistance through epigenetic mechanisms, as well as miRNAs showed that this was dependent on the IlS intestinal transcription factor DAF-16 (Nono et al., 2020). Intestinal knockdown of the histone trimethylation (H3K4) demethylase modifier ASH-2 resulted in increased oxidative stress resistance in two subsequent generations (Figure 2Dii). This effect was dependent on DAF-16 activity in the intestine through regulation of a downstream target, F08F1.3, which targeted several histone modifiers in the germline as well as being required the function of HRDE-1, a germline argonaut, which regulates miRNA activity (Nono et al., 2020). This suggests that there may be a cell nonautonomous regulation of inherited stress resistance integrating both the miRNA and IlS pathways between the intestine and germline in promoting the survival of offspring.

## The Intestine as an Integrator of Behavioral and Defense Mechanisms

**FIGURE 3:**
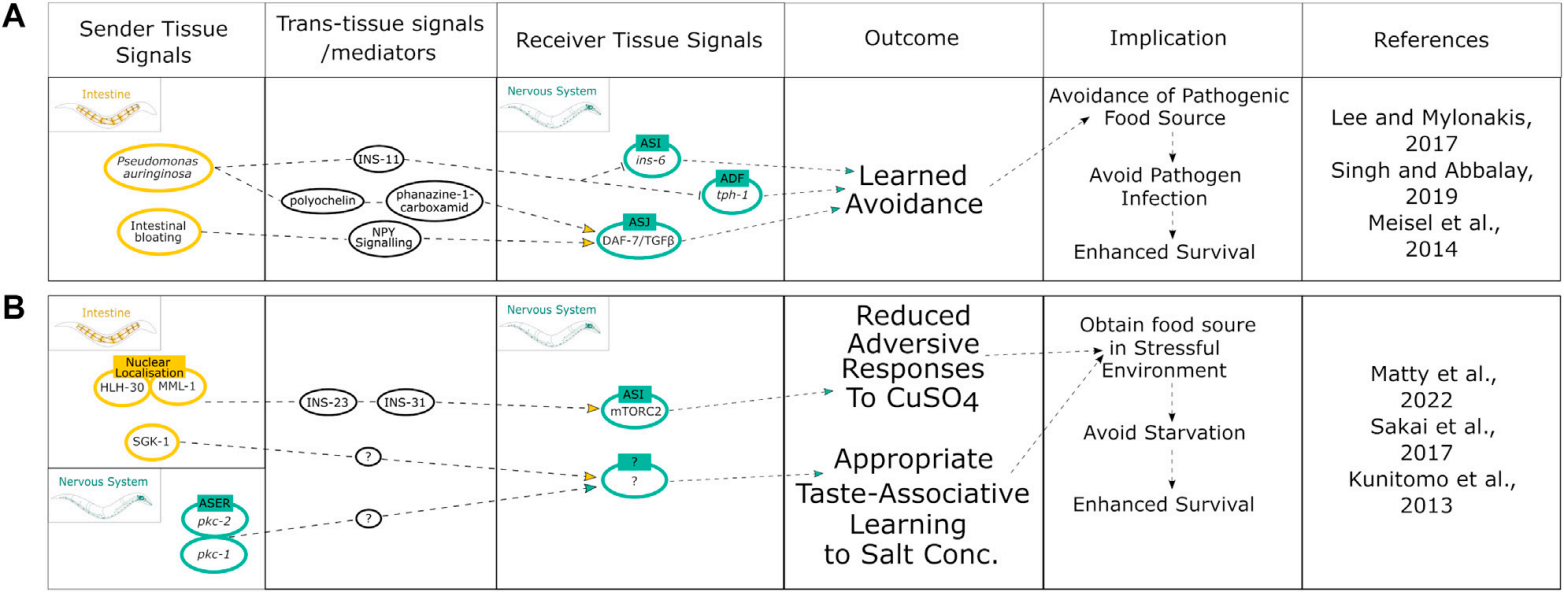
Summary of intestinal modulation of behavioral responses linked to improved stress-associated survival. **(A)** Mechanisms initiated by the intestine that modulate behavior upon pathogen infection to promote survival. **(B)** Integration of both neuronal and intestinal cues to pursue food cues by downregulating avoidance of risk stimuli to promote survival.

All examples discussed thus far have focused on how the intestine affects health- and lifespan directly. The intestine also influences neuronal cues that modulate behaviors to promote survival in response to stress stimuli and enhance proteostasis. This intestine-to-neuron crosstalk is important to drive behavioral change that can be beneficial for organismal proteostasis. Behavioral modulation is vital as a mechanism to evade pathogens and promote survival and longevity. Certain pathogenic bacteria such as *Pseudomonas aeruginosa* induce initial attraction in *C. elegans* as a food source but then lead to a subsequently learned avoidance once it has been recognized as a pathogen (Singh and Aballay, 2019). This switch to avoidance has been linked to intestinal signals. One study from 2017 points towards an intestine-derived neuropeptide, INS-11, to mediate this learned avoidance behavior through modulating IlS (*ins-6*) and serotonin signaling (*tph-1*) in ASI and ADF neurons, respectively (Figure 3A) (Lee and Mylonakis, 2017). The neuropeptide Y (NPY)-related signaling neuroendocrine pathway is activated as a result of intestinal bloating and directly targets DAF-7/TGF-β signaling in ASJ neurons creating a learned avoidance for the low-oxygen environment associated with *P. aeruginosa* (Figure 3A) (Singh and Aballay, 2019). Another study suggested that it is the release of the secondary metabolites pyochelin and phenazine-1-carboxamide, produced by *P. aeruginosa* in the intestine, which targets DAF-7/TGF-β signaling in ASJ neurons to initiate this learned avoidance behavior (Figure 3A) (Meisel et al., 2014).

Interestingly, the *C. elegans* intestine may even dictate behavioral decisions based on the level of damage sustained from a particular sensory cue (Hajdú et al., 2021). Upon exposure to toxic concentrations of benzaldehyde or diacetyl there was a cytoprotective response initiated in the intestine. This response effectively restored proteostasis in the intestine damaged by benzaldehyde but not diacetyl. The intestinal damage was suggested to be signaled from the intestine to the nervous system to confer either flexible or robust avoidance responses upon re-exposure to each chemical (Hajdú et al., 2021). Although this mechanism needs further characterization, it presents a potential role of the intestine in controlling memory formation in the nervous system to dictate future behaviors based on damage levels as a form of decision making between defensive avoidance and food searching. This balances the risk of damage with the risk of starvation to promote survival and therefore longevity. Similar findings have also implicated the intestine to integrate cues involved in “risk-versus-reward” behavioral decision making (Matty et al., 2022): *C. elegans* that had experienced food deprivation will cross a toxic barrier quicker than well-fed animals to reach a food source. The mechanism behind this elevated risk-taking was shown to involve the translocation of the transcription factors MML-1 and HLH-30 from the intestinal nuclei to the cytoplasm, as well as insulin-like peptides INS-23 and INS-31, that target the DAF-2 receptors on the ASI neurons, thereby modulating TORC2 signaling to reduce aversive chemotaxis responses (Figure 3B) (Matty et al., 2022). In addition, *C. elegans* can associate being well-fed or starved with the specific concentration of salt that either nutritional state was experienced in a manner that depends on the protein kinase PKC-1 (Gq/DAG/PKC pathway) (Kunitomo et al., 2013) and the TORC2 substrate PKC-2 in ASE neurons (Sakai et al., 2017) (Figure 3B). Moreover, the TORC2 substrate SGK-1 is required specifically in the intestine to associate low salt with starvation leading to an avoidance of low salt concentrations and attraction to high concentrations of salt, albeit the exact neuronal targets remain to be determined (Figure 3B) (Sakai et al., 2017). Thus, the intestine is required for feedback signaling to the nervous system to initiate protective behaviors that in turn benefit organismal proteostasis.

Similarly, behavioral cues initiated in the intestine can influence the responses to thermal stress by migrating away from dangerously high and low temperatures. For example, intestinal activation of the TORC2 substrate PKC-2, promotes cold-directed migration (Land and Rubin, 2017).

Together these findings indicate the intestine is able to modulate risk-taking behaviors as well as pathogen avoidance to promote organismal proteostasis.

## Discussion and Outlook

The nervous system has been the focus of trans-tissue regulation in pro survival, healthspan, and longevity signals. However, increasing knowledge points to the intestine as a key organ that feeds information towards the nervous system and other tissues in response to a variety of stressors including nutrition availability, pathogen infection, oxidative- and heat stress. The intestine has been proven to affect not only stress survival but is implicated in passing on epigenetic information to promote survival in proceeding generations. This suggests the intestine is not only a hub for health- and lifespan regulation but also a potential modulator of evolutionary adaptation to stressful environments. However, there remains much to be understood about how the intestine safeguards proteostasis across tissues and how this role can be harnessed to delay the onset of neurodegenerative diseases in patients, such as, for example, through the gut microbiome. Rats exposed to curli-producing bacteria in their gut, showed increased neuronal alpha-synuclein deposition in both gut and brain, potentially directly *via* cross-seeding of amyloid species and priming certain responses of innate immune cells in the brain, such as glial cells (Chen et al., 2016).

In mammals, the gut-brain axis plays an important role in the development of multiple age-dependent protein folding diseases, including AD, PD and amyotrophic lateral sclerosis (ALS), that is, often preceded by changes in gut microbiota. For example, a lack or decrease of certain bacterial species in the gut, can have a negative impact on PD or ALS pathogenesis in patients at an early disease stage (Jin et al., 2019; Hertzberg et al., 2022).

Interestingly, the mammalian brain senses gut stimuli *via* the passive release of hormones and other macromolecules from the gut. Epithelial sensor cells in the gut, the enteroendocrine cell or neuropod cells, can also directly connect with vagal neurons to transduce mechanical, chemical, or bacterially derived sensory signals from the gut lumen to the brain, using glutamate as a neurotransmitter [reviewed in Furness et al. (2013); reviewed in Psichas et al. (2015); Kaelberer et al. (2018)]. Thus, this allows the intestine a direct impact on neuroplasticity, brain health and proteostasis-promoting behaviors that influence overall health which is likely conserved throughout evolution.

As highlighted in this review, the intestine differentially affects different tissues, therefore further understanding of intercellular signaling events occurring between the gut and distal tissues, and in particular gut-to-neuron communication, could open exciting possibilities for future therapeutic interventions to improve brain homeostasis during aging.
